# Augmented reduction in colonic inflammatory markers of dextran sulfate sodium-induced colitis with a combination of 5-aminosalicylic acid and AD-lico™ from *Glycyrrhiza inflata*

**DOI:** 10.1080/19768354.2018.1476409

**Published:** 2018-06-02

**Authors:** Jaeyoung Cho, Hyuck-Se Kweon, Sung-Oh Huh, Ali Sadra

**Affiliations:** a Unites Inc, Chuncheon, Korea; b SynergyBio, Chuncheon, Korea; c Department of Pharmacology, College of Medicine, Hallym University, Chuncheon, Korea; d ADbiotech Co. Ltd, Chuncheon, Korea

**Keywords:** Dextran sulfate sodium, inflammation, inflammatory bowel disease, colitis, interleukin-6, tumor necrosis factor

## Abstract

The primary aim of this study was to determine whether the oral administration of AD-lico™, a functional extract from *Glycyrrhiza inflata* in combination with 5-aminosalicylic acid (5-ASA) could ameliorate the inflammatory symptoms in dextran sulfate sodium (DSS)-induced colitis in rodents. This DSS rodent model is used to study drug candidates for colitis, as part of the spectrum of diseases falling under the inflammatory bowel disease (IBD) category. Here, with oral AD-lico™ administration, there was a substantial disruption of the colonic architectural changes due to DSS and a significant reduction in colonic myeloperoxidase (MPO) activity, a marker of colitis. In the same samples, there were also reduced levels of colonic and serum IL-6 in the oral AD-lico™ treated rats. This study also addressed the possible mechanisms for AD-lico™ mediated changes on colonic inflammation markers. These included the observations that AD-lico™ dampened the IL-6 proinflammatory-signaling pathway in THP-1 human monocytic cells and reduced the TNFα-mediated upregulation of surface adhesion molecule ICAM-1 in human umbilical vein endothelial cells (HUVECs). Finally, it was shown that AD-lico™ could be combined with 5-ASA in reducing the inflammatory markers for colorectal sites affected by colitis, a first study of its kind for a combination therapy.

## Introduction

As part of the treatment strategy for patients with inflammatory bowel disease (IBD), inhibitors of tumor necrosis factor alpha (TNFα) have been shown to be effective in reducing their symptoms associated with IBD. However, 30% of patients resistant to standard therapy are also refractory to TNFα blocking agents. Also, of those that do respond, an excess of 50% stop responding after one year on those targeted agents (Ghosh and Panaccione 2010). The anti-TNFα agents also carry significant safety concerns, thus limiting their applicability. As there is the possibility of targeting alternate pathways in the inflammatory, this is an area of active research.

The conditions falling under the designation of inflammatory bowel disease (IBD) include ulcerative colitis (UC) and Crohn’s disease (CD). These are chronic conditions that are also remitting and relapsing (Strober et al. 2002). The symptoms can be debilitating for the patient and may include gastric immobility, diarrhea, fever, resulting in weight loss and colon length loss (Fiocchi 1998; Hendrickson et al. 2002). For UC, with infiltration of inflammatory cells, the colon mucosa is affected with ulceration and shortening of crypts (Strober et al. 2002). The inflammatory mediators in UC include produced reactive oxygen species (ROS) such as nitric oxide (NO) (Kolios et al. 2004; Araki et al. 2006), prostaglandins (PG) (Singer et al. 1998; Bonner 2002) and cytokines such as interleukin (IL)-6, tumor necrosis (TNF)-α and interferon (IFN)-γ (Atreya and Neurath 2005; Rojas-Cartagena et al. 2005; Ito et al. 2006).

The exact contributing factors for generation of UC remain unclear, although immune system dysfunction, heredity and dietary influences are thought to each play a role (Baumgart and Carding 2007). For targeting UC, there is ongoing research in blocking generation of inflammatory cytokines (Brasseit et al. 2016), and for animal studies, the DSS model is an exceptional preclinical model of colitis, having characteristics mimicking the human disease (Seril et al. 2003). In this model, DSS impacts the colonic epithelial barrier, which increases the colonic mucosal permeability with a resultant destruction of the crypts with inflammatory infiltration and production of pro-inflammatory cytokines, resulting in colon attributes similar to acute and chronic UC in patients (Randhawa et al. 2014).

Licorice, the root of Glycyrrhiza species, has been for centuries an herbal medicine in many Asian and European countries (Fiore et al. 2005) with reported effectiveness for ameliorating cough, peptic ulcers, constipation and various viral infections (von Boehmer and Daniel 2013; Trompette et al. 2014). Licorice is a complex mix of bioactive glycyrrhizin and flavonoids such as liquiritin, isoliquiritin, and various aglycones (Zhang and Ye 2009). The constituents of Glycyrrhiza extracts display anti-inflammatory effects in various *in vitro* systems, including lipopolysaccharide-stimulated macrophages. In these model systems, the extract constituents of glycyrrhetic acid and glycyrol have been shown to regulate the NF-κB pathway, resulting in inhibition of inflammatory cytokines such as IL-6, IL-1β, and TNFα. Glycyrol also modulates COX-2 and iNOS expression in LPS-treated macrophages (Shin et al. 2008). On whether Glycyrrhiza extracts or its constituents could regulate intestinal inflammation and to also explore the potential benefits of AD-lico™ derived from Glycyrrhiza extracts in UC therapy, we examined its effects on DSS-induced colitis in a rat model and in combination with 5-ASA. Previously, AD-lico™ was also shown to have gastric-relaxing and anti-Helicobacter effects in the rat models of these two indications (Sadra et al. 2017). The goals of this study were to evaluate the effect of AD-lico™ on colonic signs of inflammation and to investigate the role of AD-lico™ on inflammatory mediators in DSS-treated rats.

## Materials and methods

### AD-lico™ source material

AD-lico™ is a 95% ethanol extract of licorice species *Glycyrrhiza inflata* developed by ADbiotech Co. Ltd. (Chuncheon, Gangwon-Do, Korea). Briefly, powdered root from a secured source was finely ground and extracted with 95% ethanol. The extractions were at 100 g/L for 48 h at room temperature with occasional shaking, followed by filtration, and evaporation of ethanol in a water bath at 40^о^C. The remaining ethanol was removed by vacuum evaporation, leaving a fine powder. The powder was resuspended in 95% ethanol, and then serially diluted to a final concentration of less than 0.001% ethanol in water before each use. The same final solution without the initial added extract served as the negative control in the experiments.

### Rat dextran sodium sulfate (DSS) colitis model

Male Sprague–Dawley rats (Orient Bio Inc., Seongnam, Korea) weighing at 250 ± 10 g at the time of arrival were acclimated to individual cages for one week. In their cages, the animals had free access to standard rat chow and water. The cages were maintained in a temperature-controlled room at 22°C ± 2°C with a 12 h light/dark cycle. The rats were assigned to various treatment groups (*n* = 10 per treatment). Mild/moderate colitis was induced by giving rats 5% DSS (MP Bio, Santa Ana, CA, USA) dissolved in drinking water for 7 days. The treatments were initiated 4 days after the first dose of DSS and continued for 10 days. The treatments were once-a-day oral administrations of AD-lico™ at 25/50/100 mg/kg body weight (BW), 5-aminosalicylic acid (5-ASA; Sigma-Aldrich, St. Louis, MO, USA) at 50 mg/kg BW, AD-lico™/5-ASA combination at 25 and 50 mg/kg BW, respectively, and vehicle (water). The rats were checked daily for colitis development by monitoring body weight, gross rectal bleeding, stool consistency and survival. Rats were sacrificed after the final DSS administration. The protocols on treatment of the laboratory animals were approved by the Institutional Animal Care and Use Committee (IACUC) in the laboratory.

### Morphology of colon tissues

From the distal end of the colon, a length of 1 cm was removed and fixed in 4% paraformaldehyde, followed by embedding in paraffin. Morphological changes and the extent of inflammation from DSS exposure was gauged by hematoxylin and eosin staining according to the standard protocols (Fischer et al. 2008).

### Colonic myeloperoxidase (MPO) activity in the colons

Rat colons samples were first rinsed with cold PBS; they were then dabbed dry and flash frozen in liquid nitrogen. The samples were then stored at −80°C until assayed. For the MPO assay, the o-dianisidine technique was used (Krawisz et al. 1984). Briefly, after thawing, the samples were weighed and were suspended (10% wt/vol) in 50 mM potassium phosphate buffer (pH 6) with 0.5% hexadecyltrimethylammonium bromide and were then homogenized with a blender. The homogenate was sonicated for 30 s in 1 mL volume, followed by centrifugation for 10 min at 200xg at 4°C. One hundred microliters of supernatant was then mixed and incubated with 2.8 mL of 50 mM potassium phosphate, 30 mL of 20 mg/mL o-dianisidine dihydrochloride and 30 µl of 20 mM hydrogen peroxide for 10 min at 20°C. The reaction was then stopped with 30 mL of 2% sodium azide added. With a SpectraMax M2 microplate reader (Molecular Devices, Sunnyvale, CA, USA), the absorbance was read at 460 nm with the MPO activity of the sample measured with respect to control buffer. The MPO activity was designated as the enzymatic activity to lead to a change in absorbance of 1 unit/min/g of wet tissue.

### Cell culture

Acute monocytic leukemia THP-1 cells (ATCC, Manassas, VA, USA) (ATCC No. TIB­202) were maintained in RPMI­1640 medium containing 10% FBS, 0.05 mM 2­mercaptoethanol plus 50 U/mL penicillin and 50 µg/mL streptomycin at 37°C in a humidified 5% CO_2_ incubator. All the media components for these cells were from Thermo Fisher (Waltham, MA, USA). Human umbilical vein endothelial cells (HUVECs) plus antibiotics were obtained from the Japanese Collection of Research Bioresources (JCRB) cell bank (Osaka, Japan) (JCRB No. IFO50271) and were expanded in Hank’s F12 K medium (Thermo Fisher), 20% FBS (Thermo Fisher), 100 µg/mL heparin (Sigma-Aldrich) and 50 µg/mL endothelial cell growth supplement (Sigma-Aldrich). The HUVECs were kept at 37°C in a humidified 5% CO_2_ incubator. The cells were subcultured and reseeded into a suitable culture plate until their monolayer became confluent.

### Western blot analysis

THP-1 cells were first treated with various doses of AD-lico™ and also separately with Genestein (Sigma-Aldrich). The treated THP-1 cells were then activated with human IL-6. For the cell lysates, the cells were lysed with ice cold radio immunoprecipitation assay (RIPA) lysis buffer composed of 150 mM NaCl with 1% NP-40, 0.5% sodium deoxycholate, 0.1% SDS and 50 mM Tris-Cl (pH 8), containing a cocktail of protease inhibitors (Roche, Basel, Switzerland). The lysates were then centrifuged for 20 min at 13,000 × g 4°C; the supernatants were saved and their protein concentration was determined by the Bradford assay (Bio-Rad, Richmond CA, USA). Equal amounts of protein were separated by sodium dodecyl sulfate- polyacrylamide gel electrophoresis (SDS-PAGE) in 8-15% reducing gels and transferred onto polyvinylidene difluoride membranes (Millipore, Bedford, MA, USA). Following blocking with 5% non-fat milk (Difco / Becton Dickinson, Franklin Lakes, NJ, USA) in TBST (10 mM Tris, 140 mM NaCl, 0.1% Tween-20, pH 7.6), the membranes were incubated with various primary antibodies overnight at 4°C. The primary antibodies were against pERK (Thr202/Tyr204), ERK, STAT3, pSTAT3 (Tyr705), pJAK2 (Tyr1007/1008) and β-actin, which were from Cell Signaling (Danvers, MA, USA). The membranes were then washed and incubated with horseradish peroxidase conjugated secondary antibodies at room temperature for 3 h. Following washing of the membranes again with TBST, the Western blot bands were developed by an enhanced chemiluminescence (ECL) kit (Luminata Forte; Millipore) and exposure to X-ray film (Fujifilm, Tokyo, Japan).

### IL-6 in colon/serum

IL-6 levels in the serum and tissue were determined using an enzyme-linked immunosorbent assay (ELISA) (Kim et al. 2010). This involved having 96-well plates (SPL Life Science, Seoul, Korea) coated with 100 μL of anti-mouse monoclonal antibody (1 mg/mL at pH 7.4 in phosphate buffered saline [PBS]) and incubated overnight at 4°C. Following washing of the plates, the sample or rat IL-6 standard (BD Bioscience, San Diego, CA, USA) at 50 μL was added to each well and incubated at room temperature for 2 h. The plates were then washed and biotinylated anti-mouse antibody at 0.2 μg/mL was added and incubated for 2 h at room temperature. This was followed by washing and incubation of the wells with avidin-peroxidase (Sigma-Aldrich) at 37°C for 30 min. The plates were washed once more and the color development substrate, (2,2′-azino-bis [3- ethylbenzothiazoline-6-sulfonic acid]-diammonium salt) (BD Bioscience), was added to each well. The developed color was measured at 405 nm using a SpectraMax M2 microplate reader (Molecular Devices). The standard curves were generated with dilutions of rat IL-6 (BD Bioscience).

### Cell viability

Following treatment with AD-lico™ at the indicated concentrations for 24 h, cell viability was measured by a 3-(4,5-dimethylthiazol-2-yl)-2,5-diphenyl-tetrazolium bromide [thiazolyl blue tetrazolium bromide (MTT)] assay and according to the manufacturer’s protocols (Roche, Mannheim, Germany).

### ICAM-1 cell based ELISA

HUVECs at 7,000 per well in 200 µl in complete F-12 K media were plated overnight in cell culture wells. Following 16 h in the culture incubator, the cells were starved for 12 h in 2% FBS F-12 K media. The cells were then pretreated with serial dilutions of AD-lico™ stock for 3 h in culture. Subsequently, the cells were induced for an upregulation of endogenous ICAM-1 with 10 ng/mL of human TNFα (PeproTech, Rocky Hill, NJ, USA) for 8 h. Cell surface expression of ICAM-1 by HUVECs was determined using cell-based ELISA. Briefly, after treatments, the cells were washed with phosphate-buffered saline pH 7.4 (PBS) and fixed with 4% paraformaldehyde for 30 min at 4°C. Non-fat dry milk (3% in PBS) was added to the cell monolayers to reduce nonspecific binding. Cells were incubated with mouse anti-human ICAM-1/CD54 monoclonal antibodies (BBA3; R&D Systems, Minneapolis, MN, USA) overnight at 4°C after washing three times with PBS. After removal of the primary antibodies, the cells were washed with PBS and were incubated with peroxidase-conjugated goat anti-mouse secondary antibody for 30 min (Santa Cruz, Dallas, TX, USA). Thereafter, the cells were washed with PBS and exposed to the peroxidase substrate (TMB reagent; Thermo Fisher). The color development and reaction stoppage with sulfuric acid were performed according to the TMB reagent manufacturer’s protocol (Thermo Fisher). The increase in absorbance at 450 nm was measured using a SpectraMax M2 Microplate Reader (Molecular Devices).

### Statistical analysis

The data are given as mean ± SD. For determining statistical significance, student’s t test with Microsoft Excel 2010 program was used (Microsoft, Redmond, WA, USA). A *p*-value of less than 0.05 was deemed statistically significance.

## Results

### AD-lico™ inhibits the destructive morphological changes seen in DSS-treated rats

**Figure 1.**
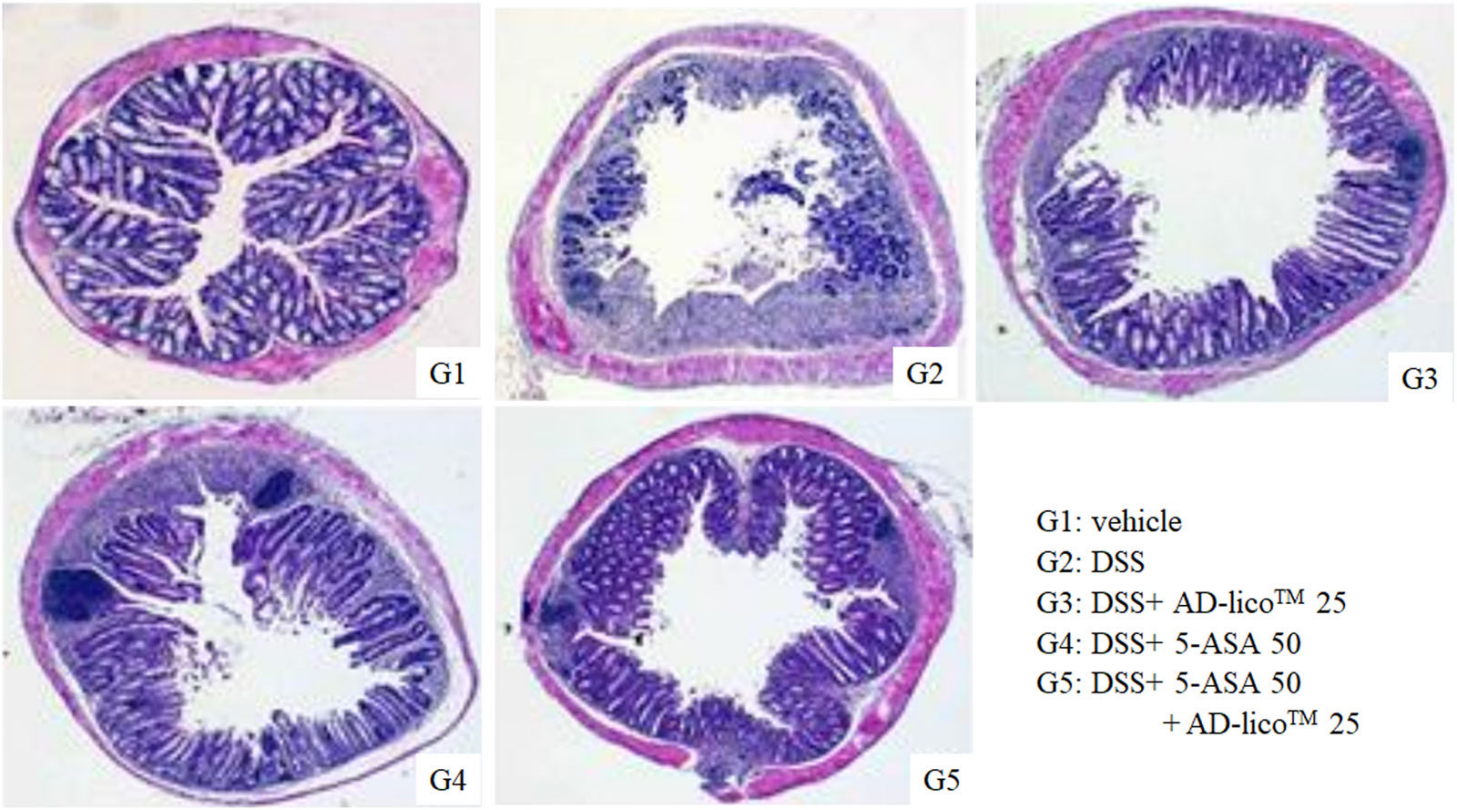
Inhibition of morphological changes in colon cross-sections due to DSS treatment. A 1-cm segment was removed from the distal end of the colon and fixed in 4% paraformaldehyde solution prior to embedding in paraffin. The degree of inflammation and morphological injury caused by 5% DSS exposure was assessed by hematoxylin and eosin staining according to the standard protocols (magnification, ×100). This is a representative of three experiments. G1, vehicle; G2, 5% DSS; G3, 5% DSS + AD-lico™ 25 mg/kg; G4, 5% DSS + 5-ASA 50 mg/kg; G5, 5% DSS + 5-ASA 50 mg/kg + AD-lico™ 25 mg/kg

Histological examination of the colonic sections assessed the intestinal inflammatory status. Microscopically, the samples from the DSS-induced colitic rats showed typical inflammatory changes in colonic architecture, such as ulceration, crypt dilation and goblet cell depletion, as well as mixed cell infiltration composed mainly of macrophages, lymphocytes, plasma cells and granulocytes (Figure 1). Conversely, histological analysis of the colons from AD-lico™-treated rats and more significantly when combined with 5-ASA showed greatly reduced numbers of infiltrating cells, degree of mucosal injury and edema (Figure 1).

### Inhibition of myeloperoxidase (MPO) activity in colon samples

**Figure 2.**
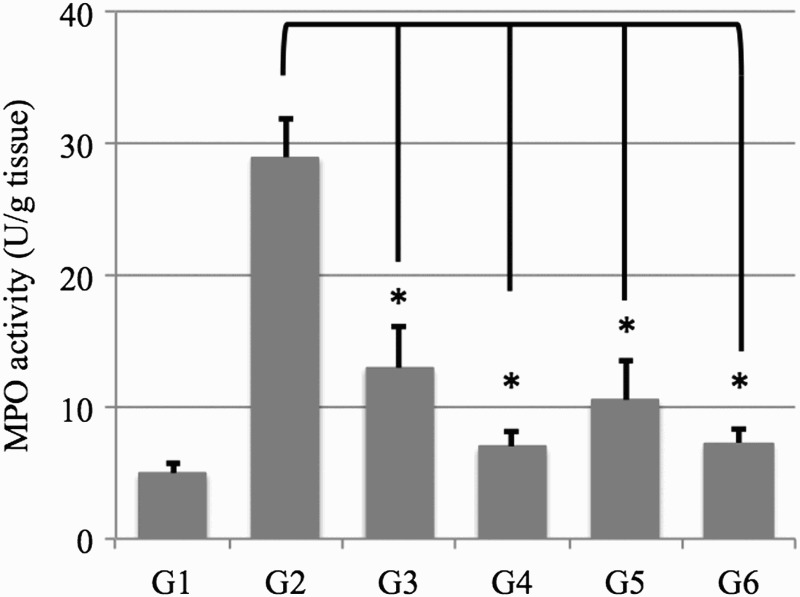
Inhibition of myeloperoxidase (MPO) activity. The rat colons were rinsed with cold PBS, blotted dry and frozen immediately in liquid nitrogen. They were then stored at −80°C until assayed for MPO activity using the o-dianisidine method. G1, normal control; G2, 5% DSS; G3, 5% DSS + AD-lico™ 25 mg/kg BW; G4, 5% DSS + AD-lico™ 100 mg/kg BW; G5, 5% DSS + 5-ASA 50 mg/kg BW; G6, 5% DSS + 5-ASA 50 mg/kg BW + AD-lico™ 25 mg/kg BW. *p < 0.05. This is a representative of three experiments.

MPO is an enzyme produced mainly by polymorphonuclear leucocytes and is associated with the degree of neutrophil infiltration in tissues. Following 7 days of DSS treatment via drinking water, MPO activity increased markedly to a level approximately four times higher than that in the control group (Figure 2). This increase in MPO activity was significantly reduced by AD-lico™ administration through day 10. The combination with 5-ASA, however, did not result in an added inhibition on top of 5-ASA alone. Because MPO activity is considered a biochemical maker of neutrophil infiltration, this result suggests that AD-lico™ exerts anti-inflammatory effects by reducing neutrophil infiltration into the colonic mucosa.

### AD-lico™ inhibits IL-6 levels in colon and serum samples from DSS treated rats

**Figure 3.**
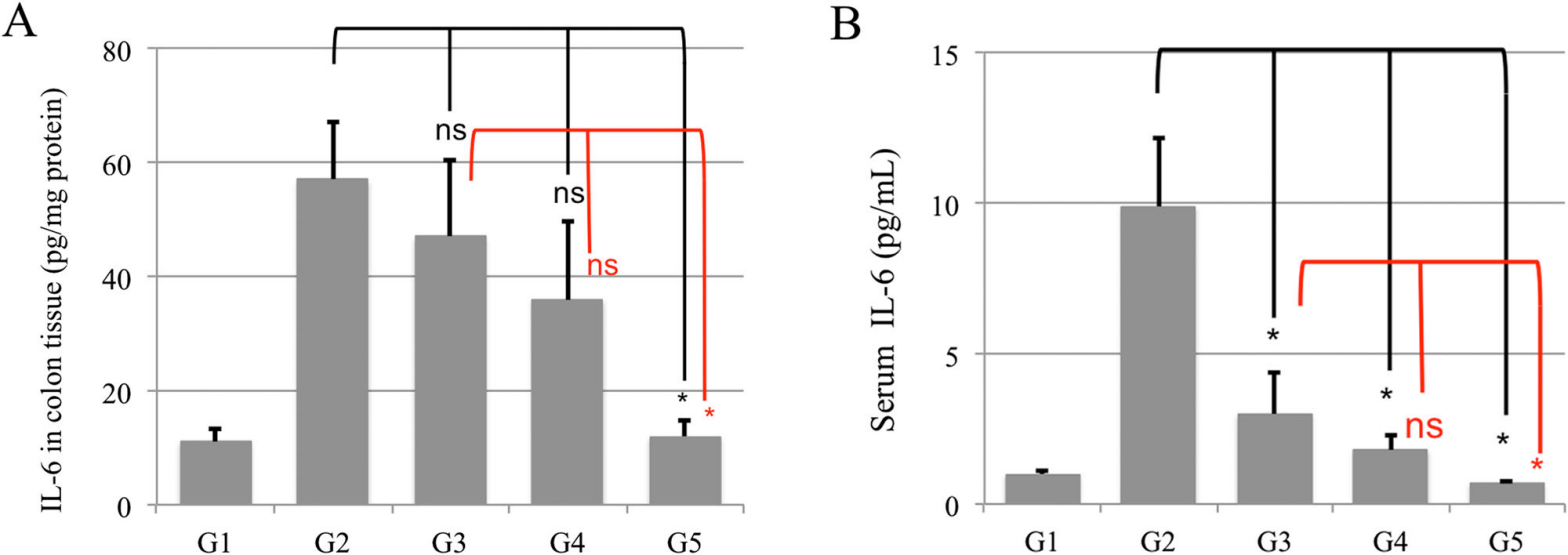
Inhibition of IL-6 levels in (A) colon and (B) serum samples from DSS treated rats. Levels of IL-6 in the serum and tissue were measured using an enzyme-linked immunosorbent assay (ELISA), as described in Materials and Methods. G1, normal control; G2, 5% DSS; G3, AD-lico™ 25 mg/kg BW; G4, 5-ASA 50 mg/kg BW; G5, 5-ASA 50 mg/kg BW + AD-lico™ 25 mg/kg BW. *p < 0.05. This is a representative of three experiments.

To determine the effect of AD-lico™ on major inflammatory cytokine expression, and IL-6 levels were determined (Figure 3). After 7 days of DSS administration, IL-6 levels both in colon tissue samples and serum from the DSS-treated rats increased significantly, compared to the control group. AD-lico™ administration and more significantly in combination with 5-ASA prevented significant increases in IL-6 levels.

### AD-lico™ inhibits IL-6 downstream signaling markers in IL-6 treated THP-1 cells

**Figure 4.**
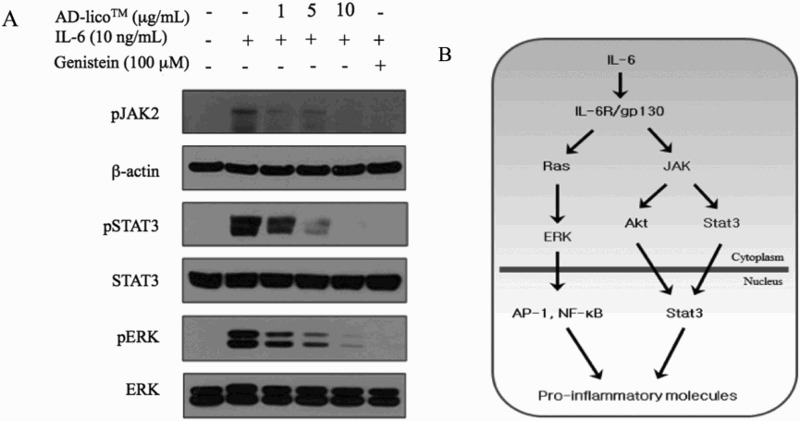
AD-lico™ inhibiting IL-6 downstream signaling markers in IL-6 treated THP-1 cells. JAK2 and ERK activation and STAT-3 phosphorylation increases due to activation of the THP-1 cells by IL-6 were dose-dependently blocked by AD-lico™ pretreatment of the cells for 2 h. Genistein, a known kinase inhibitor, served as a positive control in the experiments. This is a representative of three experiments (A). Proposed pathway components of IL-6 signaling in THP-1 cells (B).

The THP-1 cells served as a model of immune cells in response to inflammatory cytokines that can propagate the inflammatory cycle. JAK2 and ERK activation and STAT-3 phosphorylation increases due to activation of the THP-1 cells by IL-6 were dose-dependently blocked by AD-lico™ pretreatment of the cells for 2 h. Genistein, a known kinase inhibitor, served as a positive control in the experiments (Figure 4(A and B)).

### AD-lico™ blocks upregulation of surface ICAM-1 in HUVECs treated with TNFα

**Figure 5.**
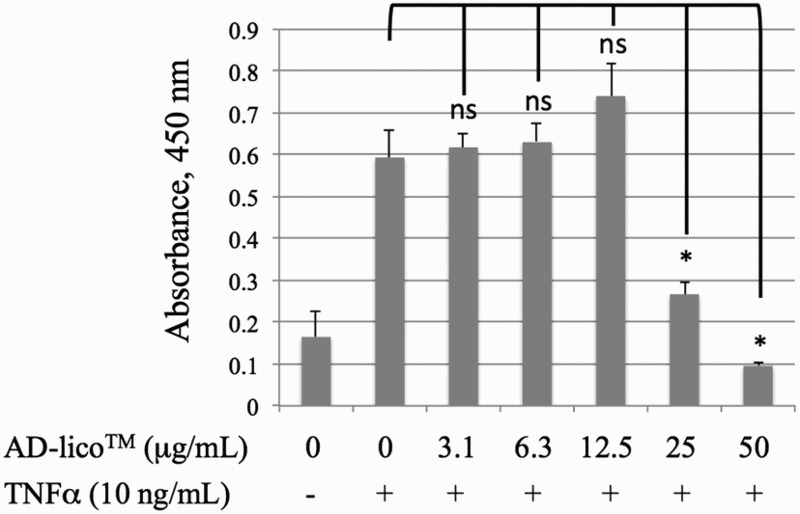
AD-lico™ blocking upregulation of surface ICAM-1 in HUVECs treated with TNFα. HUVECs at 7,000 per well in 200 µl in complete F-12 K media were plated overnight in cell culture wells. Following 16 h, the cells were starved for 12 h in F-12 K media but only containing 2% FBS. The cells were then pretreated with serial dilutions of AD-lico™ stock for 3 h in culture. Subsequently, the cells were induced for an upregulation of endogenous ICAM-1 with 10 ng/mL of human TNFα for 8 h. Cell surface expression of ICAM-1 by HUVECs was determined using cell-based ELISA. *p < 0.05. This is a representative of three experiments.

HUVECs pretreated with serial dilutions of AD-lico™ stock were induced for an upregulation of endogenous ICAM-1 with human TNFα. Cell surface expression of ICAM-1 by HUVECs was determined using cell-based ELISA. Endothelial cells treated with TNFα showed significant increases in the expression of ICAM-1 (Figure 5). This induction, which caused a greater than 3.5-fold increase in control expression, was decreased in a dose-dependent manner by AD-lico™ pretreatment.

## Discussion

AD-lico™ is derived from the root of *Glycyrrhiza inflata*, a licorice plant member, known to have a variety of medicinal effects. These include anti-microbial, anti-inflammatory and anti-atherosclerotic activities (Fiore et al. 2005). For its anti-inflammatory effects, AD-lico™ inhibits ICAM-1 upregulation in the target endothelial cells. It is known that ICAM-1 is involved in interactions with the beta-2 class of integrins, leading to inflammatory responses from target cells in various scenarios (Chang et al. 2010). For example, for lipopolysaccharide (LPS)-activated macrophages, blocking ICAM-1 also blocks PGE2 synthesis along with NO production and increased expression of iNOS (Chang et al. 2010). Also, licorice extract treatment significantly reduced plasma levels of NO and TNFα in an LPS septic shock model (Kang et al. 2005). In a previous study, it was also shown that an ethanolic extract of licorice had strong anti-inflammatory effects (Kim et al. 2006). For these reasons, we tested AD-lico™ for activity in treating the inflammatory responses in IBD.

For the *in vivo* efficacy studies, we chose the DSS-induced colitis rodent model, which displays symptoms similar to those seen in the human version of UC that include ulceration of the mucosa and shortening of the colon; these are accompanied by presence of blood in the feces, diarrhea and overall body weight loss (Rufo and Bousvaros 2006). The DSS-induced colitis animal model also has advantages in having reproducibility in terms of course of onset and severity (Okayasu et al. 1990; Cooper et al. 1993).

Although AD-lico™ is a desirable candidate as an anti-inflammatory agent, its inhibitory effect on ICAM-1 expression in HUVEC remained unknown. In this study, we demonstrated that AD-lico™ inhibits TNFα-activated ICAM-1 protein levels, potentially leading to the suppression of immune cell adhesion to endothelial cells. ICAM-1 is considered to play a key role at the early stages of inflammatory response in increasing leukocytes adhesion and transmigration across the vascular endothelial cells. We observed that AD-lico™ inhibited ICAM-1 protein expression, suggesting AD-lico™ reducing monocytes adhesion by its inhibition of ICAM-1 expression. The upregulation of ICAM-1 with TNFα involves the MAPK signaling pathway with increases in the activity of transcription factors, such as NF-κB and AP-1, which in turn increase the expression of various adhesion molecules with ICAM-1 being chief among them (Hoefen and Berk 2002). Among the various MAPK pathways, the JNK pathway has been identified as responsible for activating AP-1 in response to TNFα-induced ICAM-1 expression (Ventura et al. 2003). AP-1 is composed of c-Jun and c-Fos, and JNK phosphorylates the activity domains of c-Jun (Karin 1995).

In vascular endothelial cells, NF-κB transcriptionally regulates TNFα-stimulated ICAM-1 expression, and the activity of NF-κB is mediated by homodimeric or heterodimeric combinations of NF-κB family proteins. At its resting state, NF-κB is present in association with its cytoplasmic inhibitor, IκB. Following cellular activation by an inflammatory cytokine, such as TNFα, IκB is rapidly phosphorylated and degraded, leading to the translocation of activated NF-κB from the cytoplasm to the nucleus (Hacker and Karin 2006). This may, at least in part, account for the mechanism of AD-lico™ exerting its anti-inflammatory effects.

In summary, our results demonstrate that AD-lico™, singly or more effectively in combination with 5-ASA, reduces the colitis phenotype in the DSS rat model. We also provided a mechanistic evidence of regulation of the inflammatory pathway both in the macrophage model cells and in primary endothelial cells. These findings provide for oral AD-lico™ as being potentially salutary in treatment of colitis associated with IBD, used singly or in combination with active sulfasalazine class of drugs such as 5-ASA.
